# Precision Medicine in Aortic Anastomosis: A Numerical and Experimental Study of a Novel Double-Sided Needle

**DOI:** 10.3390/jpm11121385

**Published:** 2021-12-20

**Authors:** Danae G. Manolesou, Georgia Korompili, Dimitris Davazoglou, Andreas M. Lazaris, Dimitrios Schizas, Despina Sanoudou, Theodore Liakakos, Constantinos Tsioufis, Theodore G. Papaioannou

**Affiliations:** 1 Biomedical Engineering Unit, First Department of Cardiology, Hippokration Hospital, Medical School, National and Kapodistrian University of Athens, Mikras Asias 75, 115 27 Athens, Greece; ktsioufis@hippocratio.gr (C.T.); thepap@med.uoa.gr (T.G.P.); 2 Institute of Nanoscience and Nanotechnology, NCSR Demokritos, Neapoleos 27, 153 41 Athens, Greece; g.korompili@inn.demokritos.gr (G.K.); d.davazoglou@inn.demokritos.gr (D.D.); 3 Department of Vascular Surgery, Attikon Hospital, Medical School, National and Kapodistrian University of Athens, Mikras Asias 75, 115 27 Athens, Greece; andreaslazaris@hotmail.com; 4 First Department of Surgery, Laiko Hospital, Medical School, National and Kapodistrian University of Athens, Mikras Asias 75, 115 27 Athens, Greece; schizasad@gmail.com (D.S.); theodlia@med.uoa.gr (T.L.); 5 Clinical Genomics and Pharmacogenomics Unit, 4th Department of Internal Medicine, School of Medicine, National and Kapodistrian University of Athens, Mikras Asias 75, 115 27 Athens, Greece; dsanoudou@med.uoa.gr; 6 Center for New Biotechnologies and Precision Medicine, School of Medicine, National and Kapodistrian University of Athens, Mikras Asias 75, 115 27 Athens, Greece; 7 Center of Basic Research, Biomedical Research Foundation of the Academy of Athens, 4 Soranou Ephessiou, 115 27 Athens, Greece

**Keywords:** vascular surgery, aorta, experimental devices, surgical needle

## Abstract

Background: Hand-sewn anastomosis is a crucial part of aortic reconstruction surgery and significantly affects its outcome. The present study presents a novel, bidirectional surgical needle aimed to improve aortic anastomosis in terms of speed and ease of use. Our objective was to assess the efficacy of the new design in comparison with the conventional needle. Methods: A series of simulations were conducted with COMSOL software in order to perform a fatigue comparative analysis between the new and the conventional needle design. Ease of penetration into a piece of polydimethylsiloxane was evaluated. Lastly, the prototype was tested under in-vitro conditions in comparison with the conventional needle. Results: Based on fatigue analysis, the new needle design improves durability, provided the two tips are equally used. The polytetrafluoroethylene coating improves penetration into the tissue by 7% to 17%, while electropolishing improves penetration up to 19%. When using the novel needle design, the average anastomotic task completion time was significantly reduced by 22% and the overall distance of hand movements was significantly reduced by 20%. Conclusions: The proposed design exhibited a shorter anastomotic time and seems promising in relation to ease of use and simplicity of the anastomotic technique it introduces.

## 1. Introduction

Aortic aneurysms, the second most common disease of the aorta after atherosclerosis [1], may affect any part of the vessel; however, abdominal aortic aneurysms are the most frequent, with a prevalence of 1.3% (United Kingdom) to 5% (United States) [2,3]. Despite the widespread use of the endovascular approach, open reconstruction of the diseased aortic segment remains an integral part of aortic aneurysm treatment, especially for patients anatomically unfit for endovascular repair [4] or younger ones with a long life expectancy and lower perioperative risk (4916 procedures in 2013, as documented in United States Medicare datasets [5]). Anastomosis, the surgical restoration of the continuity of the vessel, is a crucial part of the procedure and significantly affects its outcome. Since Alexis Carrel’s pioneering work on vascular anastomosis [6], suturing of the aortic end to the graft has been the gold standard. However, hand-sewn anastomosis is technically demanding; the stability of the surgeon’s posture, hand, needle and needle holder, the needle angle, and entry/exit pathway curve are only few of the factors affecting the anastomosis’ performance [7]. Technical errors while performing the anastomosis can cause early postoperative bleeding at the site, which may lead to anastomotic aneurysm formation with a prevalence of 2% to 29% [8]. Furthermore, hand-sewn anastomosis can be time-consuming, even for an experienced surgeon. Extended anastomotic time translates to extensive aortic cross-clamping time, which is defined as greater than 30 min for thoracoabdominal aneurysm repair [9] and greater than 50 min for abdominal aneurysms [10], can provoke severe hemodynamic changes in various organs [11], and is related to worse clinical outcomes [12,13].

Several alternative methods have been suggested incorporating the use of ringed grafts, stents, staplers, couplers, or adhesives, however, none of them have successfully replaced the hand-sewn technique in everyday clinical practice. Previous research on the efficacy of such devices in end-to-end anastomosis of the aorta has shown that, although they offer a faster anastomosis and satisfactory short-term mortality rates, they are inferior in terms of tensile strength compared with the hand-sewn technique [14,15].

Herein, we present the design, development, and preclinical assessment of a novel, bidirectional needle for use in aortic anastomosis, aimed at increasing surgical precision and effectiveness. The proposed needle has two tips that can both be used for penetration to the tissue. This new design aims to eliminate complex maneuvers related to the rotation of the tip of the needle using the needle-holder and the forceps alternately. Several studies have previously capitalized on the use of double-sided needles, such as in plastic surgery [16,17,18,19,20], orthopedic surgery [21], and wound closure [22,23]. However, to the best of our knowledge, there are no studies describing the use of a bidirectional needle in aortic surgery. To assess the clinical potential of the new needle in comparison with the conventional approaches, we performed a series of in silico and in vitro tests, including: (a) fatigue usage factor simulations and (b) experiments measuring the ease of insertion and (c) exploratory testing evaluating the speed and ease of use. Our findings suggest a heightened clinical value, contributing towards the advancement of precision medicine approaches in cardiovascular surgery. The proposed new anastomotic technique aspires to be more precise, more user-friendly, and less time-consuming for the surgeon.

## 2. Materials and Methods

**Figure 1:**
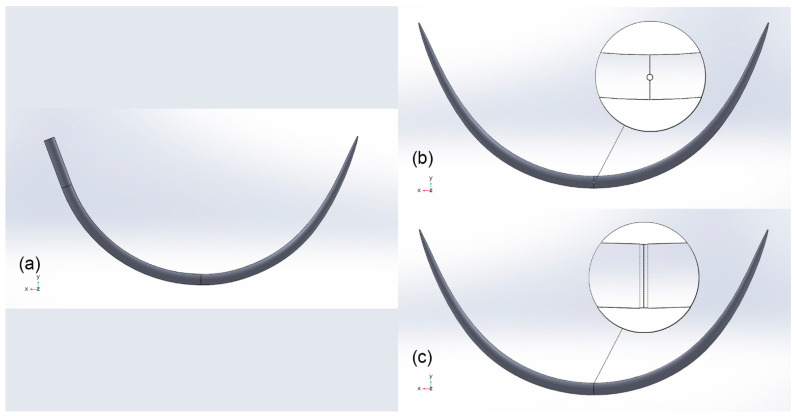
3D digital model of (**a**) the conventional needle, (**b**) the first iteration of the new needle with the suture hole parallel to the needle axis, and (**c**) the second iteration of the new needle with the suture hole vertical to the needle axis.

Needle Design. The proposed needle design incorporates two tips in order to eliminate the need for the 180° rotation of the needle during anastomosis. The suture hole is placed in the middle of the length of the needle body. Placing the hole closer to one of the tips was avoided as it would complicate the grasping of the needle with the needle-holder. The precise design geometry was based on the conventional 1/2 curved needle. The conventional needle was observed under a standard optical microscope to accurately measure the needle thickness and a Scanning Electron Microscope (SEM) (Jeol FESEM 7401f, Japan) to investigate the minimum surface of the tip. The model of the new needle was designed in SolidWorks software with 800 μm maximum body diameter and a 120 μm diameter at each one of the two tips. With regards to the suture hole, we chose to assess two options; the first version had a hole oriented parallel to the needle axis (Figure 1b), while the second had a hole oriented vertical to the needle axis (Figure 1c). We also assessed three iterations of the hole size for each version: 100 μm, 200 μm, and 300 μm.

Simulations. The structural changes proposed in the new design are expected to improve the procedure of aortic anastomosis, but also to affect the needle’s durability due to the hole hosting the thread that is located in the middle of the needle core. Through simulations—conducted with the COMSOL Multiphysics’ solid mechanics module—we aimed to estimate if the proposed design is less prone to failure in comparison to the conventional needle, when the same fatigue analysis is performed. The fatigue analysis comprises of the application of a defined load, which does not provoke fracture for a high number of cycles (high cycle analysis), precisely 10^3^ cycles. We selected to perform a stress-based fatigue analysis by employing the Findley criterion [24]:

$$max\left(\frac{\Delta \tau }{2}+k\;\cdot \;\sigma_{n}\right)\leq f$$

where k is the normal stress sensitivity coefficient and f is the Findley limit factor of the applied pressure. Both k and f are material parameters. Δ*τ* is the maximum shear stress range on a plane and *σ_n_* is the largest normal stress on the same plane. The analysis results in the fatigue usage factor $F_{us}$ defined as the ratio:

$$F_{us}=\frac{max\left(\frac{\Delta \tau }{2}+k\;\cdot \;\sigma_{n}\right)}{f}$$

**Figure 2:**
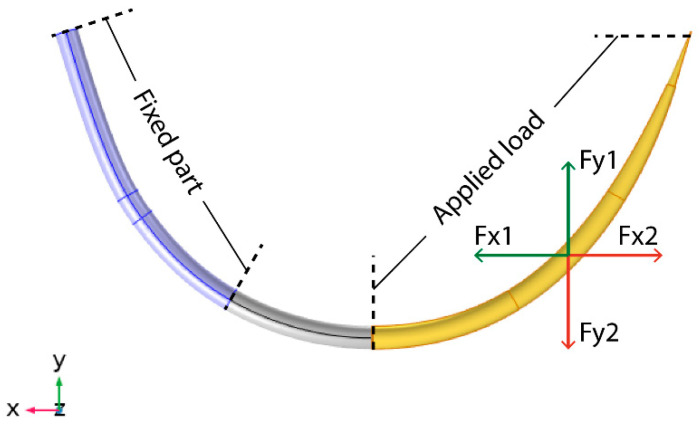
The conventional needle design with the basic simulation aspects of the loading scenario applied: 1/3 of the needle is held fixed, simulating the position of the needle holder while 1/2 of the needle body is subjected to the applied load cases, defined by the combination of two vertical forces each time.

The fatigue usage factor is a dimensionless metric, defined as the ratio of the stress induced in the needle when a specific load is applied over the f parameter. Thus, the closer the $F_{us}$ value is to 1, the closer the component is to the fatigue limit. To perform the fatigue analysis, a scenario of repetitive load cycles was selected. For high cycle analysis, a load smaller than the fracture limit was applied for 10^3^ cycles. Thus, a force of up to 1 N was selected to be applied in both x- and y- directions (Figure 2), with an alternating direction between load cases 1 and 2. The selected force did not cause a fracture in the needle body. The force was applied on the one half of the needle, while on the other half, a part corresponding to 1/3 of the total needle length was held fixed, simulating the grasp of the needle-holder. The repeated load cases are reported in Table 1, while all parameters referring to the selected material properties used in the simulation are presented in Table 2, corresponding to the properties of stainless steel 308L. The same model and simulation scenario were repeated for all of the tested designs; different hole sizes, ranging 100 μm, 200 μm, and 300 μm, and orientations, parallel and vertical to the needle axis, were tested.

**Figure 3:**
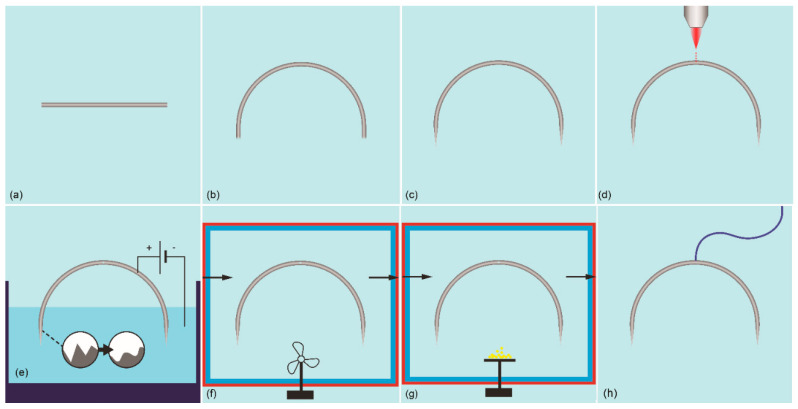
Needle prototype manufacturing process: (**a**) 308L wire cut at appropriate length, (**b**) bending, (**c**) sharpening of needle tips, (**d**) laser drilling of the suture hole, (**e**) electropolishing of the prototype surface, (**f**) LPCVD of tungsten on the surface of the prototype, (**g**) PTFE coating, and (**h**) attachment of the suture.

Prototype Construction. The fabrication process (Figure 3) was based on well-established manufacturing processes used for surgical needle fabrication, as described in the literature [25,26]. A piece of thin (d = 800 μm) stainless steel wire was manually bent at the desired curvature (r = 10 ± 2 mm) and was cut according to the length of the conventional 1/2 circle surgical needle (26 ± 3 mm). Subsequently, the two end points were sharpened by a drill loaded with a deburring stone tip. The suture hole was created afterwards by drilling a 200 μm diameter with a 30 W fiber laser beam. The surface of the needle was then polished with the standard electro-polishing process [27]. The electrolyte was composed of phosphoric acid, glycerin, and deionized water. The process was accomplished by the application of 10 V between the anode and the cathode with a current limit set at 500 A, and it was performed twice per needle to cover the entire surface of it, not just the tip region. The aim of this process was to smooth the outer surface of the needle, remove any residues from the laser drilling process, protect the needle from corrosion [27], and enhance the ease of penetration into the tissue.

In patients requiring aortic reconstruction, the aortic tissue is often stiff due to inflammatory aneurysmal tissue or extensive aortic calcification [28,29]. Therefore, a hardening step of the needle is imperative to increase the needle’s durability when inserted multiple times into the aortic tissue during surgery. Low pressure chemical vapor deposition (LPCVD) [30] of tungsten by the pyrolysis of tungsten hexacarbonyl (W[CO]6) vapors [31] was selected to provide a strengthened coating on the outer surface of the needle. This method was preferred over the thermal treatment hardening of the entire needle body of the needle, so as to avoid an increase in the needle’s brittleness. Depositions were carried out in a cold wall LPCVD reactor with a quartz chamber equipped with a graphite susceptor externally heated by three tungsten-halogen lamps of 1000 W each, on which the needles were placed. The precursor vapors (W[CO]6) were produced in a saturator held at 90 °C and were transferred into the reaction chamber with a regulated N_2_ flow of 30 sccm. Depositions were carried out at 550 °C and at a pressure of 0.5 Torr for 30 min. It is known from previous studies [32] that with the above-described method, tungsten layers containing carbon and oxygen impurities (WxCyOz) were deposited on the needles. To enhance the smoothness of the surface of the needle, a thin film of polytetrafluoroethylene (PTFE) was deposited, as described in the literature [33].

As a last step, a 6-0 polypropylene suture was passed through the suture hole. In turn, the needle body at the point of the suture hole was compressed bilaterally to secure the suture in place.

Prototype characterization process. Following the construction of the novel needle prototype, we conducted a series of experiments on the characterization of the manufacturing process and on the assessment of the new needle in comparison with the conventional one.

**Figure 4:**
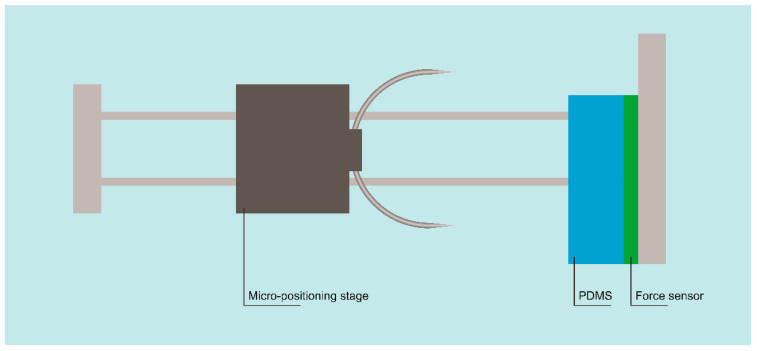
Schematic illustration of the set-up used for tissue penetration testing.

Initially, we examined the ease of needle insertion into a flexible 7700 μm thick piece of polydimethylsiloxane (PDMS). PDMS—with a base elastomer/curing agent ratio of 7:1—was selected as a low-cost material to simulate the tissue’s mechanical behavior. A cantilever beam weighing scale force sensor was combined with an HX711-24-bit A/D and a microcontroller to measure the load applied on the PDMS when the needle was inserted into it with a resolution of 5 mg. The force sensor was placed on the backside of the PDMS part and the needle was fixed on an automated micro-positioning stage that forced the needle to penetrate the PDMS with a constant displacement rate of 500 μm/min. The experimental setup is illustrated in Figure 4. The experiment resulted in the determination of the slope in the curve of the force vs. the micro-stepping. The higher the slope, the more difficult the penetration of the needle tip into the PDMS substrate. Thus, a comparison of the slope in the corresponding curves between the conventional needle, the constructed needle after the electropolishing process, and the constructed needle after the LPCVD process was conducted to give an estimation of the needle surface roughness. Secondly, we assessed the needle tip sharpness under a scanning electron microscope (Nanotechnology and Microsystems Laboratory, NCSR “Demokritos”, Athens, Greece).

**Figure 5:**
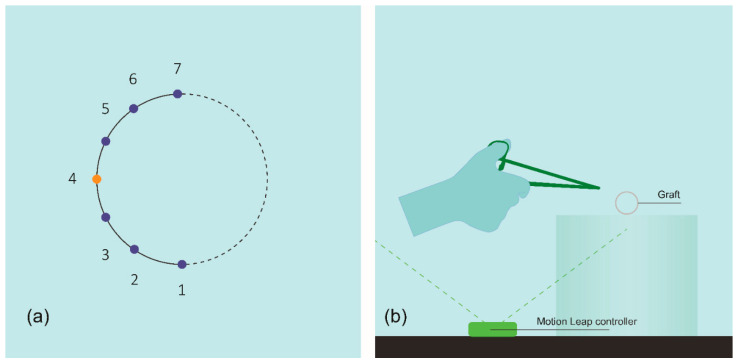
(**a**) Schematic of the anastomotic task performed at the in-vitro prototype testing: at the fourth stitch the needle direction is changed. (**b**) Schematic of the prototype testing set-up. The plexiglass box hosting the graft is 15 cm in height, so the anastomosis is performed inside the vision field of the infrared sensor.

Prototype testing. To assess the feasibility of the concept, the new needle was tested at an in vitro level. An adequate number of prototypes were constructed following the same manufacturing process for the new and the conventional needle design. Furthermore, a plexiglass box was built to host the graft (Gelsoft, Vascutek); one part of the graft was stabilized on the horizontal plane of the box to simulate the aortic segment on which the proximal anastomosis was performed, while the other part was left “free” to be sutured to the former. Two experienced vascular surgeons and two vascular surgeons in training performed half of an end-to-end anastomosis, applying seven stitches, as shown in Figure 5a. We chose the specific task as a simulation of one of the most common, over-and-over anastomotic techniques used in clinical practice today. Overall, the anastomotic task was completed 14 times using the new design and 14 times using the conventional one. The experimental set-up is shown in Figure 5b.

We recorded the time needed to complete the task and calculated the distance the right hand of each surgeon travelled in space. To capture the surgeon’s hand movements while performing the task, we used the Brekel Hands Pro software (see Supplementary Video S1) in combination with the Leap Motion infrared sensor, a hand-tracking device that has recently been used in intraoperative image interaction research [34]. We chose to assess the collected data for the point of the right palm of the surgeon. To calculate the distance traveled, the Euclidean metric was utilized, which is defined as follows:

$$d\left(A,B\right)=\sqrt{{\left(\chi_{B}-\chi_{A}\right)}^{2}+{\left(y_{B}-y_{A}\right)}^{2}+{\left(z_{B}-z_{A}\right)}^{2}}$$

where A and B are two points in three-dimensional space. Because the point of the right palm of the surgeon passes through *n* (where *n* ∈ N) points in space, we compiled an algorithm that calculated the distance travelled by adding the Euclidian metrics between each point. More specifically, if $q_{1}\ldots q_{n},\;n\in N$ are the points through which the point of the right palm travels, the overall distance was calculated as follows:

$$\Sigma d\left(q_{i},\;q_{1}\right)=d\left(q_{i},q_{i-1}\right)+\Sigma d\left(q_{i-1},q_{1}\right)$$

where:

i=2…n , n ∈ N

and:

$$d\left(q_{i},q_{i-1}\right)=\sqrt{{\left(\chi_{q_{i}}-\chi_{q_{i-1}}\right)}^{2}+{\left(y_{q_{i}}-y_{q_{i-1}}\right)}^{2}+{\left(z_{q_{i}}-z_{q_{i-1}}\right)}^{2}}$$

Furthermore, tracking of the points of the hand was realized in a local system, in other words from Child to Parent point; more specifically, the position of the right palm in space is given in relation to the position of the right wrist and the position of the right wrist in relation to the right elbow. Thus, we defined that the coordinates $\chi_{q_{i}},y_{q_{i}},\;z_{q_{i}}$ of each point $q_{i}=(\chi_{q_{i}},y_{q_{i}}\;,\;z_{q_{i}}),\;i=1\ldots n\;$, n ∈ N, through which the right palm passed, are calculated as follows:

$$\chi_{q_{i}}=x_{Rpalm}+x_{Relbow\;}+x_{Rwrist}\;y_{q_{i}}=y_{Rpalm}+y_{Relbow\;}+y_{Rwrist}\;z_{q_{i}}=z_{Rpalm\;}+z_{Relbow\;}+z_{Rwrist}$$

The procedure was also documented on a camera to investigate the changes in the anastomotic process that the novel design induced. We assumed that the steps needed to remount the needle and excessive manipulations with the needle-holder were eliminated. Overall, we are interested in the ease of handling of the new needle, the time required to complete the anastomosis, and the distance traveled while performing the task.

## 3. Results

### 3.1. Needle Fatigue Factor Estimation

**Figure 6:**
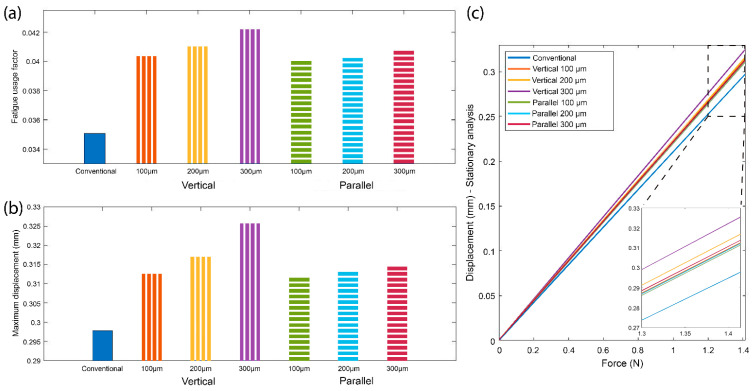
COMSOL simulation results on (**a**) the fatigue usage factor for all types of needles tested: the conventional one containing one tip and the proposed design containing two tips with the hole hosting the suture placed in the middle with either vertical or parallel direction with respect to the needle axis. (**b**) The maximum displacement exhibited by the needle tip in the steady state for the load cases examined in the simulation. (**c**) The steady state displacement with respect to the absolute applied force.

The application of the loading scenario described earlier results in the estimation of the fatigue usage factor for all of the tested needle designs (Figure 6). It is clearly depicted that the suture hole placement in the middle of the needle body affects the needle durability by increasing the fatigue usage factor in both cases of the hole axis direction. When the hole axis is placed parallel to the needle axis direction, the fatigue factor increases by 16%—for the 300 μm hole diameter—compared to the fatigue usage factor of the conventional needle, where no hole is present. This increase is slightly higher (up to 20%—for 300 μm hole diameter) for the case of the vertical hole axis, which was anticipated due to the direction of the force in the applied loading scenario. A force applied in the z axis (see Figure 2) would have affected more the “parallel” hole design than the “vertical” one, however, this type of scenario was excluded from simulations as it was considered to be rather rare in the use of the needle when penetrating into the tissue.

The increase in the fatigue factor was tested for different hole diameter sizes for both parallel and vertical hole axis designs, in comparison to the conventional design. Again, the vertical hole direction design seemed to be more affected by the hole size compared to the horizontal hole direction design. The results are also accompanied by the corresponding values of the maximum displacement of the needle tip in each case for the steady state condition. The absence of the needle material in the space of the suture hole results in the possibility of the needle to bend more when the same load is applied.

To interpret the above results, we should take into consideration that the new needle—irrespective of the hole orientation and size—will be affected less than the conventional needle with a single tip, due to the alternate use of the tips during the anastomosis. This assumption is based on the way the needle is mounted on the needle-holder [35]. During the insertion of one tip into the tissue, the needle-holder is placed at the 1/3 of the needle length, on the side of the non-inserted tip of the needle (see Figure 2 “fixed part”). In this way, the non-inserted tip of the needle is kept fixed and should not be affected by the stresses on the inserted tip of the needle. Thus, an increase in the fatigue factor of each tip of the new needle up to 50% is acceptable for achieving at least the same durability as the conventional needle.

### 3.2. Ease of Penetration into the Tissue and Tip Sharpness

**Figure 7:**
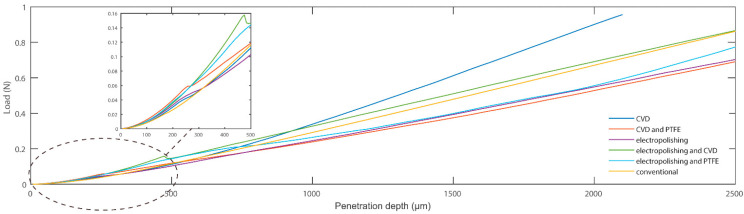
The curve corresponding to the measured load on the PDMS substrate during needle penetration vs. the penetration depth of the needle. The slope of the curve is indicative of the ease of penetration into the selected material, simulating the tissue.

To estimate the ease of needle penetration, we measured the load applied on the PDMS substrate while the needle was inserted into it. We present separate measurements for (a) the conventional needle (Foosin Medical Supplies) and the new needle prototype after (b) the electropolishing process; (c) the electropolishing and LPCVD process; (d) the electropolishing and PTFE coating process (e) the electropolishing, LPCVD, and PTFE coating processes. The slope of the curves illustrated in Figure 7 indicates the ease of penetration into the material simulating the tissue; the lower the slope, the lesser the load applied on the needle during insertion into the PDMS, at the same depth. The slope is extracted through a MATLAB curve fitting algorithm. The percentage of difference to the slope of the conventional needle was calculated for all cases. The novel needle after the tip sharpening process through electropolishing seemed to penetrate the tissue easier than the conventional needle—the slope of the curve was approximately 19% lower. The process of PTFE deposition was expected to improve penetration, as all data corresponding to needles with PTFE coated surface present improved penetration into PDMS, with the range of slope reduction being 7–17% in comparison with the conventional needle. The LPCVD process resulted in a needle worse than the conventional in terms of surface roughness, unless PTFE deposition was performed afterwards. Thus, in the final prototype manufacturing process we opted for both electropolishing and PTFE deposition processes to be accomplished together with Tungsten deposition through LPCVD. Lastly, slight irregularities could be observed at the beginning of the measured load curve (penetration depth 0–500 μm), which could be attributed to defects of the tip sharpening process.

**Figure 8:**
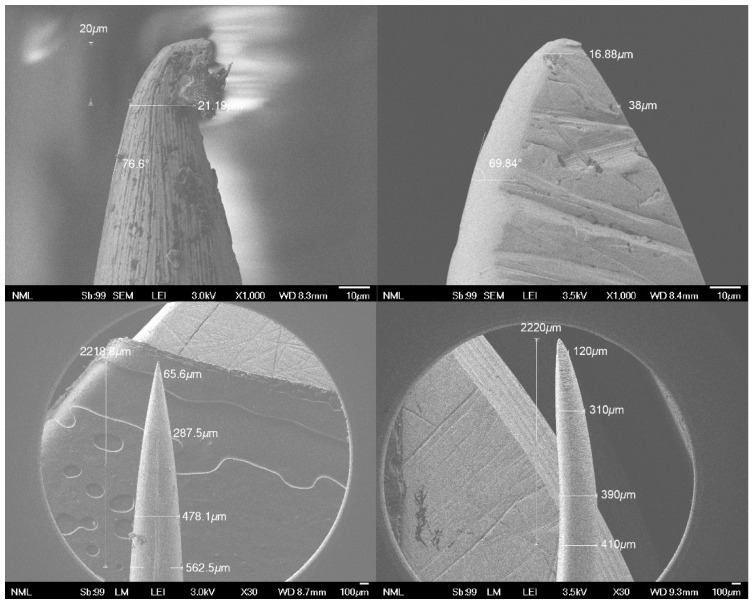
SEM images of the tip of the conventional (**left**) and the new needle prototype (**right**) at ×1000 (**top**) and ×35 (**bottom**) magnification.

Scanning electron microscope examination of the two needles at ×1000 magnification (Figure 8) showed that, at approximately 20 μm from the tip, the width of the fabricated needle was slightly wider, forming a blunter tip, in comparison with the conventional, commercial needle (38 μm and 21.19 μm, respectively). However, the scanning photographs also revealed a significant irregularity at the very edge of the conventional needle tip, probably due to the specific honing process applied by the manufacturing company. Furthermore, scanning electron microscope examination of the two needles at ×30 magnification showed that, at 2220 μm from the needle tip, the width of the fabricated needle was narrower in comparison with the conventional one (410 μm and 562.5 μm, respectively), demonstrating a relatively longer tapered point (Figure 7).

### 3.3. Prototype Testing

**Figure 9:**
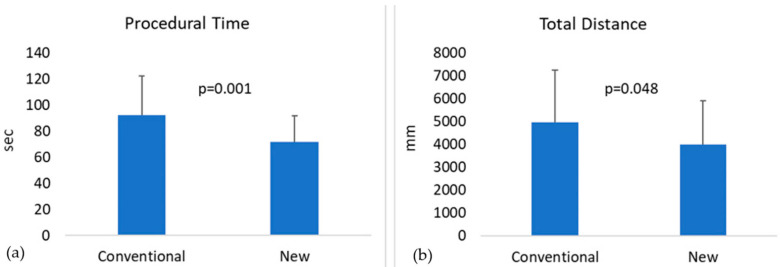
(**a**) The average time for the completion of the anastomotic task with the conventional and the new needle, and (**b**) the average distance travelled by the right palm of the surgeon while performing the task, using the conventional and the new design.

For the anastomotic samples recorded (Table 3), we performed a paired, non-parametric Wilcoxon Signed-Rank test. It was found that the average task completion time was significantly reduced by 22% with the new needle design compared to the conventional—71.6 ± 20.0 vs. 92.0 ± 30.2 s, respectively (Figure 9a). Moreover, the overall distance of the hand movements was significantly reduced by 20% using the new needle compared to the conventional—3992.3 ± 1927.1 vs. 4977.3 ± 2274.9 mm, respectively (Figure 9b).

## 4. Discussion

The global death rate from aortic disease increased from 2.49 per 100,000 in 1990 to 2.78 per 100,000 in 2010 [1]. More specifically, aortic aneurysms account for about 13,000 deaths and 55,000 hospital discharges per year (United States) [36]. Endovascular aneurysm repair has gained widespread adoption; however, several randomized controlled trials (EVAR1, DREAM, and ACE) have found higher re-intervention rates in comparison with open surgical repair, which remains an integral part of aortic aneurysm treatment options [2,37]. Hand-sewn anastomosis of the treated aortic segment is a crucial part of open aortic surgery. Suturing the graft to the aorta has been the gold standard to restore the continuity of the vessel; however, it is a challenging and time-consuming process. Previous research on the development of an alternative aortic anastomotic device has been significant, but none of the suggested devices have been finally adopted in everyday clinical practice. Devices such as ringed grafts, stents, staplers, couplers, and adhesives [24,25,26,27,28,29,30,31,32,33] have been considered in an effort to improve aortic anastomosis, and their use under experimental conditions has been examined. Through an elaborate meta-analysis [14], our team assessed the efficacy of these experimental devices in comparison with hand sewn anastomosis for the outcomes of anastomosis time and point of anastomosis rupture. The study shows that experimental alternative anastomotic devices significantly decrease the time needed to perform the anastomosis, but they are inferior in terms of the tensile strength of the hand-sewn technique, which might be credited to its ability to be adapted to almost any tissue condition that may be encountered. The results enhance the findings of Garitey et al. [38], who found that an anastomosis made of clips or stent tears more easily than the conventional one. Garitey et al. also found that a graft-to-graft anastomosis performed with an intestinal stapler has equivalent strength to the hand-sewn one; however, it was not possible to apply it to human cadavers, because the staples were tearing the tissue.

Among the experimental device types, three of them were also tested in clinical settings: the ringed graft, the anastomotic stent, and a few commercial intestinal staplers. The ringed graft device was the most widely used; however, its application was finally challenged. Another review study [15] showed that although the device was designed to form automated ringed anastomosis on each side of the graft, a successful two-side ringed anastomosis was performed for only half of the patients. Several researchers have previously highlighted the difficulties during the placement of a ringed graft, such as mismatch between the ring diameter and aneurysm neck or the inability to insert the ring due to extensive calcification of the vessel, which make the device use significantly dependent on the anatomy of the patient.

Hand-sewn anastomosis is a method that is able to adapt to almost any aortic tissue condition, which contributes to its effectiveness, strength, and resistance to rupture. However, it can be time-consuming and demands a high level of technical skill. Through on-site observation, we noted that 1/4 of the steps of the anastomotic procedure are related to rotation of the tip of the needle using the needle-holder and the forceps alternately. Apart from contributing to a high learning curve, these complex maneuvers are repeated for the placement of the stitches and often damage the tip of the needle, or result to needle break. Furthermore, remounting of the needle mostly happens outside of the surgical field. By eliminating the aforementioned steps, the proposed double-sided needle aims to offer a faster and easy to use anastomotic technique, while maintaining the benefits of hand-sewn anastomosis.

Regarding the feasibility of the approach, our preliminary results show that the placement of the suture in the middle of the needle body will increase the fatigue usage factor (by up to 16% and 20% for a 300 μm hole in parallel and vertical orientations, respectively). Nevertheless, the new design is expected to exhibit a higher durability, provided the two tips are equally used. Thus, it can be argued that an increase in the fatigue factor of each tip up to 50% is acceptable for achieving at least the same durability as the conventional needle. One limitation of our numerical analysis is the assessment of the stresses induced in the needle tip during penetration, which could not be accomplished as it requires precise experimental measurement of the aortic tissue stiffness.

In relation to prototype characterization, scanning electron microscopy assessment showed that the constructed prototype exhibited a slightly blunter proximal part of the tip in comparison with the standard needle, while the distal part of the tip was narrower in comparison with the conventional needle. Nevertheless, ease of penetration testing demonstrated that PTFE coating improves penetration into the tissue by 7% to 17%, while electropolishing improves penetration up to 19%.

Lastly, exploratory testing of the device in graft-to-graft anastomosis showed that the concept is promising in relation to speed and ease of use. Comparing its efficacy with the conventional needle, our results exhibit that the average time to complete the anastomotic task was significantly less when using the new needle. In addition, the distance travelled by the right hand of the surgeon was significantly shorter while using the new needle. As observed through video recording, the proposed design eliminates remounting maneuvers outside of the surgical field, while it also offers the option to rotate the needle from backhand to forehand without applying changing position manipulations with the needle-holder. There is limited research on surgical technique analysis and evaluation. However, Hosseinpour et al. published a study [35] documenting needle manipulation techniques that avoid remounting of the needle outside of the surgical field, which is considered a step that slows down the progress of the suture line. The new needle enables the surgeon to perform the anastomosis in a “mattress technique” manner, alternately using a backhand stitch without changing position (see Supplementary Video S2). There are a few studies [39,40,41,42] reporting the use of continuous mattress technique using the standard needle in aortic anastomosis, in order to achieve speed and durability. We argue that the application of the novel needle in that technique, rather than in the conventional “over-and-over” technique, will further shorten and simplify the procedure; however, this is to be proven by extended assessment of the device. One possible concern relates to the diameter of the hole made on the tissue, as the distal part of the needle passes through the tissue in parallel with the proximal part of the suture. This issue can be tackled at a later stage of the device optimization process by engineering the suture specifically to start with a smaller diameter and continue with an adequately larger one.

## 5. Conclusions

In this study, we designed, developed, and assessed the potential of a novel, bidirectional needle for use in aortic surgery. The proposed design exhibited a shorter anastomotic time and seems promising in relation to the ease of use and simplicity of the anastomotic technique it introduces. Further research is needed to assess its sealing effectiveness at an in vivo level.

## Figures and Tables

**Table 1:** Load cases defining the forces applied on the needle repeatedly in order to perform fatigue analysis.

Load Case	Force Fx (N)	Force Fy (N)
**Load Case 1**	0:0.1:1	0:0.1:1
**Load Case 2**	0:−0.1:−1	0:−0.1:−1

**Table 2:** 308L stainless steel properties used in the fatigue analysis simulations.

Material Parameter	Stainless Steel 308L Value
**Poisson’s Ratio**	0.30
**Density**	8 g/cm^3^
**Young’s Modulus**	200 GPa
**Normal Stress Sensitivity Coefficient *k***	0.23
**Limit Factor *f***	579 MPa

**Table 3:** The time for task completion (in seconds) and the distance travelled by the right hand of the surgeon (in mm)—14 individual prototypes of the new design and 14 individual prototypes of the conventional design were used.

Time		Distance	
New	Conventional	New	Conventional
126.0	79.8	6501.7	1532.6
122.4	91.2	6108.9	5302.4
84.6	81.0	7277.7	6734.8
75.0	70.8	3181.4	4874.2
120.0	84.0	7374.2	4847.5
91.8	86.4	3790.8	2820.9
74.4	64.2	3563.4	3315.7
135.0	80.4	5165.1	1389.8
82.8	69.6	3613.1	3484.3
63.0	28.8	2624.1	2339.2
127.8	88.2	8583.7	6115.7
88.2	84.0	7833.3	7486.4
64.8	64.8	1858.3	3295.0
32.4	28.8	2206.0	2353.6

## Supplementary Materials

The following are available online at https://www.mdpi.com/article/10.3390/jpm11121385/s1, Video S1: Short demonstration of hand-tracking using the Brekel Hands Pro software, Video S2: Forehand to Backhand demonstration using the new needle prototype.
